# Progress in Glaucoma

**Published:** 1900-10-20

**Authors:** 


### PROGRESS IN GLAUCOMA.

An interesting, and we think somewhat important, case is reported by Mr. Work Dodd,[1] in which he removed the superior cervical ganglion of the sympathetic on each side in the hope of giving relief to a case of chronic glaucoma. This operation has been advocated by Dr. Jonnesco, of Bucharest, who has reported seven cases, and claims that it has been followed by a permanen t fall in the ocular tension in all cases, very energetic contraction of the pupil, even in those cases which had already been iridectomised, disappearance of periorbital pains and cephalalgia when these existed, disappearance of the attacks in the irritative glaucoma cases, and very marked and permanent improvement of the sight in the cases where optic atrophy was not complete. Dr. Jonnesco believes glaucoma to be central and not peripheral in origin, and considers that, although by suppressing the superior cervical ganlion the origin of the trouble is not removed, the ways of transmission are destroyed. He thinks that this operation should be especially successful in chronic irritative glaucoma and in simple chronic glaucoma. At any rate, as Mr. Work Dodd says, it is an undoubted fact that iridectomy has been found to fail in most cases of the chronic form of glaucoma, and that we are just now in a state of peculiar uncertainty as to the best treatment of this most troublesome and painful disease.

Mr. Work Dodd's patient was a woman aged 62 years, who had been under treatment, off and on, for about fourteen years at the Royal Free Hospital, during which time her vision had been steadily and slowly diminishing, and she had never been free from pains in the eyes and "all parts" of the head, while exacerbations, by which extreme suffering was caused, came on at intervals of about six months. The operation described was evidently a difficult and severe one, and one not to be lightly undertaken, especially in view of the fact that it must usually require to be done on both sides; but we are free to confess that, with a steadily progressing case of chronic irritative glaucoma, much is justifiable.

[1] Lancet, October 13.

